# Rheumatoid factor IgM autoantibodies control IgG homeostasis

**DOI:** 10.3389/fimmu.2022.1016263

**Published:** 2022-10-21

**Authors:** Antonella Nicolò, Timm Amendt, Omar El Ayoubi, Marc Young, Stephanie Finzel, Makbule Senel, Reinhard E. Voll, Hassan Jumaa

**Affiliations:** ^1^ Institute of Immunology, Ulm University Medical Center, Ulm, Germany; ^2^ Department of Rheumatology and Clinical Immunology, University Medical Center Freiburg, Freiburg, Germany; ^3^ Department of Neurology, Ulm University Medical Center, Ulm, Germany

**Keywords:** autoantibodies, rheumatoid factor, affinity, homeostasis, autoimmunity

## Abstract

Rheumatoid arthritis is an autoimmune disease characterized by joint inflammation due to autoantibodies targeting multiple self-proteins. Most patients with poor prognosis show elevated titers of IgM antibodies specifically binding to IgG. Such autoreactive antibodies are referred to as rheumatoid factor (RF). However, their biological function and contribution to disease progression remains elusive. We have recently shown that autoreactive antibodies are present in healthy individuals and play an important role in regulating physiological processes. This regulatory mechanism is determined by the class and affinity of the autoreactive antibody, as low-affinity autoreactive IgM neutralizes the recognized autoantigen while high-affinity IgM protects its autoantigen from degradation. Here, we show that RFs possessing a high affinity and mono-specificity to IgG have a stabilizing effect on IgG, whereas low-affinity polyreactive RFs neutralize IgG *in vivo*. These results suggest that autoreactive IgM antibodies recognizing IgG play a crucial role in regulating IgG homeostasis and that a disbalance between IgM-mediated IgG degradation and stabilization might affect the onset and progression of autoimmune diseases. Consequently, restoring this balance using low-affinity anti-IgG IgM might be a promising therapeutic approach for autoimmune diseases involving autoreactive IgG.

## Graphical Abstract

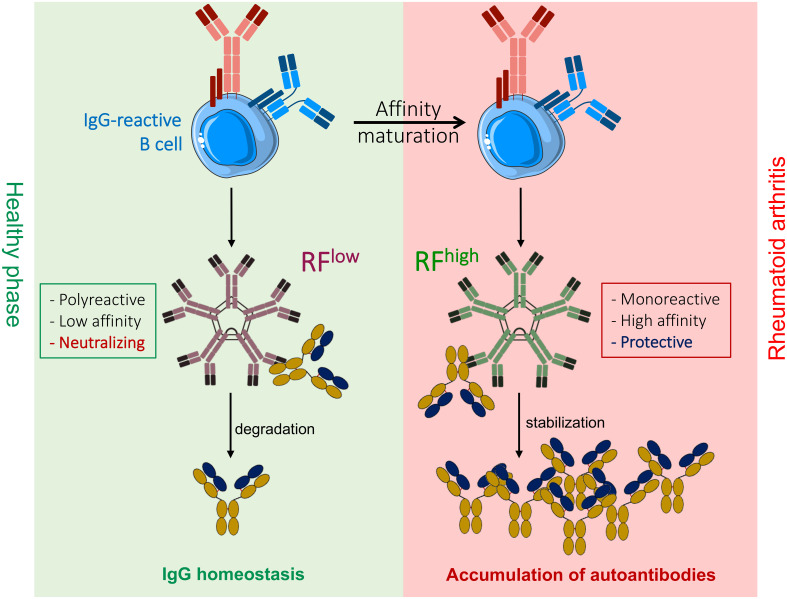

## Introduction

The process of antibody generation leads to the formation of virtually infinite antigen binding sites by random rearrangement of gene segments, namely Variable (V), Diversity (D) and Joining (J) segments (1–3). The random nature of antibody specificity generation ensures the recognition of a nearly unlimited variety of antigens but inevitably leads to the generation of self-reactive specificities (4, 5). The majority of early B cells possess autoreactive BCRs (4) and it is believed that the highly autoreactive cells are eliminated from the repertoire by central tolerance, which induces receptor editing by secondary Immunoglobulin (Ig) gene recombination thereby altering the specificity of the autoreactive B cells (6–8). If receptor editing fails to replace the autoreactive specificity, the respective autoreactive B cells are eliminated by clonal deletion (8–10). If autoreactive B cells escape from central tolerance, they are thought to be functionally silenced as mature B cells by anergy in the periphery (8, 11). Defects in the elimination of autoreactive B cells are thought to lead to the occurrence of autoimmune diseases such as rheumatoid arthritis (RA) or systemic lupus erythematosus (SLE), which are characterized and diagnosed by the presence of characteristic autoantibodies (12–15).

In sharp contrast to the concept of deleting autoreactive specificity, we have recently used insulin as typical autoantigen to show that autoreactive antibodies are normally present in healthy individuals and might play a role in regulating physiological processes beyond pathogen recognition. Moreover, we found that high affinity autoantibodies of the IgM class protect their cognate antigen and that this class of autoantibodies is generated in the course of adaptive immune responses to autoantigens (16, 17). Therefore, we refer to this mechanism as adaptive tolerance and to the high affinity IgM, which protects its cognate antigen, as protective-regulatory IgM (PR-IgM). Adaptive tolerance proposes that high affinity autoreactive IgM are generated as memory immune response to autoantigens. Thus, in contrast to primary IgM, which is generated during early phases of the immune response, PR-IgM is monospecific and binds with high affinity to the respective antigen.

Therefore, self-recognition by a specific class of high affinity mono-specific IgM autoantibodies is important for self-protection and prevention of autoimmune destruction (18). While this scenario is in contrast to the proposed general removal of autoreactive specificities, it suggests that unrestricted diversity is not only required for the elimination of potentially infinite pathogens but also for the specific protection of self by the generation of memory PR-IgM.

Rheumatoid Factor (RF) is one of the first discovered and most studied autoantibodies, already described in the late 1940s as a class of Ig that can bind the Fc portion of IgG (12, 14, 19–21). Among the different RF isotypes, IgM-RF is the most clinically used to estimate disease prognosis in rheumatoid arthritis (RA), a chronic autoimmune disease marked by chronic synovitis with infiltration of B and T cells in the synovial membrane of the joints. However, the biological function of RF in disease pathogenesis remains largely unknown (14, 20, 22).

An important characteristic of RA is the presence of anti-citrullinated protein antibodies (ACPA) causing inflammation in the synovia (23, 24). Here, removal of the amino group ( ${\text{NH}}_{3}^{+}$ ) of arginine residues by protein arginine deaminases (PAD4) generates citrullinated proteins mainly localized in joints (25, 26). Binding of ACPA-IgG to citrullinated proteins seems to lead to the deposition of immune complexes in the joints thereby activating innate immune cells and initiating inflammation.

In this scenario, it is conceivable that RFs acquire pathogenic properties through formation of immune complexes with autoreactive ACPA-IgG antibodies, thereby causing inflammation by stimulating the secretion of proinflammatory cytokines (20, 27). Interestingly, RA patients are classified into RF positive (RF+) and RF negative (RF-), where the presence of RF indicates a poor prognosis (23, 24).

RF antibodies are mainly linked to RA, nonetheless studies of RF production and incidence have shown that circulating RFs can be found in healthy individuals (19, 28–30). Interestingly, RF antibodies which have been studied in RA patients are characterized by extensive somatic mutation and possess high antigen-binding affinity and specificity for IgG acquired during the process of affinity maturation (19, 20, 28, 29, 31). On the contrary, RFs found in healthy individuals closely resemble natural autoantibodies, a class of autoantibodies with restricted epitope specificity, mainly encoded by germline variable gene segments. As such, the majority of natural autoantibodies is polyreactive and binds self-molecules with low antigen-binding affinity (32–34). Similarly, RFs in healthy individuals show no evidence of affinity maturation and isotype switching, suggesting low antigen-binding affinity for IgG (19, 20).

Though the presence of autoantibodies in normal healthy population has always been related to defective clearance of autoreactive specificities, there are evidences suggesting that autoantibodies are retained in circulation as they play a specific role in maintenance of physiological homeostasis (16, 18, 19, 32, 33, 35, 36).

In full agreement, our recent work showed that blood glucose levels are tightly regulated by insulin-specific autoantibodies of the IgM and IgG class (17, 18). In particular, we demonstrated that insulin-specific IgG neutralizes circulating insulin in wild-type (WT) mice, while PR-IgM autoantibodies with high specificity and affinity for insulin, generated in the course of secondary immune responses, play a protective role on circulating insulin by preventing IgG-induced insulin degradation. Conversely, primary insulin-specific IgM antibodies (primary IgM, pIgM) which are polyreactive and possess low affinity for insulin, lead to target destruction and thus to higher blood glucose levels (16–18).

Based on our data with insulin-specific antibodies and on published reports showing that the absence of affinity maturation distinguishes RFs found in healthy individuals from RFs of RA patients, we propose that RFs of RA patients act as PR-IgM and protect their target IgG. Here, we investigate the role of RFs possessing different affinities as potential regulators of IgG homeostasis.

## Materials and methods

### Mice

8- to 15-week-old female C57BL/6 mice were used in all animal experiments reported in this study. For antibody stability experiment, 20-50 µg antibodies (as indicated in details in figure legend for each experiment) were injected intravenously (i.v.) into the lateral tail vein and blood was collected at indicated time points to obtain serum.

For blood glucose monitoring experiments 100 µg anti-insulin IgG or anti-insulin IgM were injected i.v. into the lateral tail vein and blood was collected at indicated time points to obtain serum.

Animal experiments were performed in compliance with the guidelines of German law and approved by the responsible regional board Tübingen, Germany under the license 1484. All mice used in this study were either bred and housed within the animal facility of Ulm University under specific-pathogen-free conditions or obtained from Charles River at the age of 6 weeks.

Antibody specificity, host/isotype, conjugate clone, class, supplier catalog number:

Anti-human CD20 (Rituximab, human IgG1, SelleckChem); Rheumatoid Factor Concentrate (Lee Biosolutions #508-27), Human IgM (unlabeled, SouthernBiotech, #0158L-01), RF^low^(human IgM, homemade, sequence of heavy chain and light chain from (37)- RC1); anti-Insulin IgG (purified from IVIg, see below); total serum IgM (isolated from healthy donor serum, see below); anti-insulin IgM^high^ and anti-insulin IgM^low^ (human IgM, homemade, sequence from (38), germline reversion was achieved using the online available tool IMGT^®^ V-Quest).

### HEK293-6E cell culturing and antibody production

HEK293-6E cells were cultured in FreeStyle F17 expression media (Invitrogen) supplemented with 0,1% Kolliphor ^®^ P188 (Sigma-Aldrich) and 4mM L-Glutamine (Gibco Life Technologies). Transfections were performed according to the manufacturer’s instructions. Briefly, cells were transfected using Polyethylenimine (Polysciences) with two pTT5 plasmids encoding heavy and light chain of the antibody of interest (total 1 µg DNA/ml of culture). 24-48 hours post transfection cells were fed with Tryptone N1 (TekniScience Inc #19553) to a final concentration of 0,5%.

Harvesting was performed 120 hours post transfection. Antibodies were purified using HiTrap^®^ IgM columns (GE Healthcare, Sigma-Aldrich) as described below.

### Antibody purification and pulldown of total serum IgM

For IgM purification from human serum, IgG depletion was performed by incubating the samples with Protein G Sepharose beads (GE Healthcare, Sigma-Aldrich) according to manufacturer’s instructions.

For IgM purification from IgG-depleted human serum and from HEK293-6E cell supernatant, HiTrap^®^ IgM columns (GE Healthcare, Sigma-Aldrich) were used according to the manufacturer’s protocol and eluates were dialyzed overnight in 300-fold sample volume 1x PBS. Quality control of the isolated immunoglobulins was addressed *via* SDS–PAGE stained with Coomassie-brilliant blue R-250 (BIO-RAD) and the quantification of eluted proteins was assessed *via* ELISA.

### Isolation of antigen-specific immunoglobulins from IVIg

Streptavidin bead columns (Thermo Scientific, #21115) were loaded with 20 µg biotin-insulin (ibt biosystem) or biotin-IgG (Southern biotech, in house-labelled). IVIg preparation was incubated for 90 min at room temperature to ensure binding of antigen-specific antibodies to the beads. Isolation of the antibodies was performed by acidic pH-shift using the manufacturer’s elution and neutralization solutions. Quality of the isolated immunoglobulins was examined *via* SDS–PAGE stained with Coomassie-brilliant blue R-250 (BIO-RAD) and ELISA. For further in vivo experiments, the isolated antibodies were dialyzed overnight in 300-fold sample volume 1x PBS.

### Enzyme-linked immunosorbent assay

96-Well plates (Nunc, ThermoScientific) were coated either with 10 µg/ml anti-human IgM or anti-human IgG-antibodies (SouthernBiotech) or with 10 µg/ml human IgG (SouthernBiotech) or with 2,5 µg/ml calf thymus dsDNA (Rockland) or with 2,5 µg/ml native insulin (Sigma-Aldrich). Blocking was done in 1% BSA blocking buffer (SERVA). Serial dilutions of 1:3 IgM or IgG antibodies (SouthernBiotech) were used as standard. The relative concentrations stated as arbitrary unit (AU), were determined *via* detection by alkaline phosphatase (AP)-labeled anti-IgM/anti-IgG (SouthernBiotech). The p-nitrophenylphosphate (pNPP; Genaxxon) in diethanolamine buffer was added and data were acquired at 405 nm using a Multiskan FC ELISA plate reader (Thermo Scientific). All samples were measured in duplicates.

Antibody specificity, host/isotype, conjugate clone, class, supplier catalog number: anti-human IgM (goat, IgG, unlabeled, polyclonal, SouthernBiotech, #2020-01); anti-human IgG (goat, IgG, unlabeled, polyclonal, SouthernBiotech, #2040-01); human IgM (unlabeled, SouthernBiotech, #0158L-01); human IgG (unlabeled, SouthernBiotech, #0150-01); anti-human IgM (mouse, AP, monoclonal, SouthernBiotech, #9020-04); anti-human IgG (Goat, AP, polyclonal, SouthernBiotech, #2040-04).

### HEp-2 slides and fluorescence microscopy

Kallestad HEp-2 slides (BIO-RAD, #26101) were used to asses reactivity of purified homemade IgM or pulldown serum IgM to nuclear antigens (ANA). Approximately 10 µg per sample were applied onto the HEp-2 slides. Anti-IgM-FITC (Biolegend, #314506) was used for detection of ANA-IgM. Stained HEp-2 slides were analyzed using fluorescence microscope DMi8 (Leica) and Leica Application Suite X (LAS X) software (Leica).

### Monitoring of blood and urine glucose levels

AccuChek (Roche Diagnostics, Mannheim) blood glucose monitor was used to measure blood glucose levels of mice. Blood was taken from the lateral tail vein from *ad libitum* fed mice and transferred onto sterile test stripes. Glucose levels were measured in mmol/l at hours stated in the figures for each mouse per group.

### SDS–PAGE, Coomassie

Samples were separated on 10–12% SDS–polyacrylamide gels incubated with Coomassie-brilliant blue R-250 (BIO-RAD) for 45 min and subsequently de-stained.

### Macrophage phagocytosis assay

Cells were isolated from the peritoneal cavity of 8- to 15-week-old female C57BL/6. After isolation, cells were cultured overnight at 37°C at a density of 200.000 cells per well on microscope slides (epredia #X1XER310B).

For immune complex preparation, 20 µg of antibody, 20 µg of antigen and 20 µg of dsDNA were pre-mixed and incubated for 1 hour at 37°C. Immune complexes were incubated with macrophages for 1 hour at 37°C. Anti-human IgG-Alf-647 (Jackson ImmunoResearch, #109-605-003) was used for detection at fluorescent microscope.

Images were acquired by using Leica TCS SP8 confocal microscope with 400x magnification. Images were analyzed using ImageJ software.

### Healthy donors and patients samples

Healthy donor blood samples were obtained *via* the Deutsches Rotes Kreuz Ulm (DRK). Samples were divided into young (18-35 years) and aged (above 55 years old) according to their age. Sera was obtained by Pancoll gradient centrifugation.

Sera from multiple sclerosis patients were provided by the Biobank of the Rehabilitationskrankenhaus of the University Hospital Ulm (RKU).

Sera from rheumatoid arthritis (RA) patients were provided by the Department of Rheumatology and Clinical Immunology, Medical Center – University of Freiburg. Samples were biobanked at the ImmRheum biobank (partner of FREEZE biobank).

### Bio-layer interferometry

Bio-layer Interferometric assays (BLItz device, FortéBio) were used to determine the affinity of antigen-antibody interactions (39). Here, we used insulin-specific IgM or RF-IgM and insulin-bio (ibt biosystem) or human IgG-bio (labeled using LYNX Rapid Biotin Antibody conjugation kit, BIORAD) as targets. Targets were loaded onto streptavidin biosensors (FortéBio). Binding affinities of IgM to insulin or IgG were acquired in relative wavelength shift in nm. Subsequently, the calculated affinity value (K_a_) was used to determine the dissociation constant (K_D_): K_D_ = 1/K_a_. The following protocol was used during the measurements: 30-s baseline, 30-s loading, 30-s baseline, 120-s association, 60-s dissociation. For buffering of samples, targets, and probes, the manufacturer’s sample buffer (FortéBio) was used.

### Statistical analysis

Graphs were created and statistically analyzed by using GraphPad Prism (version 8.0) software. Data sets were analyzed by a D’Agostino and Pearson omnibus normality test and/or a Shapiro-Wilk normality test to determine if the values were normally distributed. If one of the datasets was not normally distributed or the sample number (*n)* was too small to perform normality tests, non-parametric tests were used. The numbers of individual replicates or mice (*n*) and statistical tests used to calculate *P*-values are stated within the figure legends. *P*-values < 0.05 were considered to be statistically significant (*P < 0.05; **P < 0.01; ***P < 0.001, ****P < 0.0001).

## Results

### Recombinant low affinity anti-insulin IgM neutralizes insulin *in vivo*

**Figure 1:**
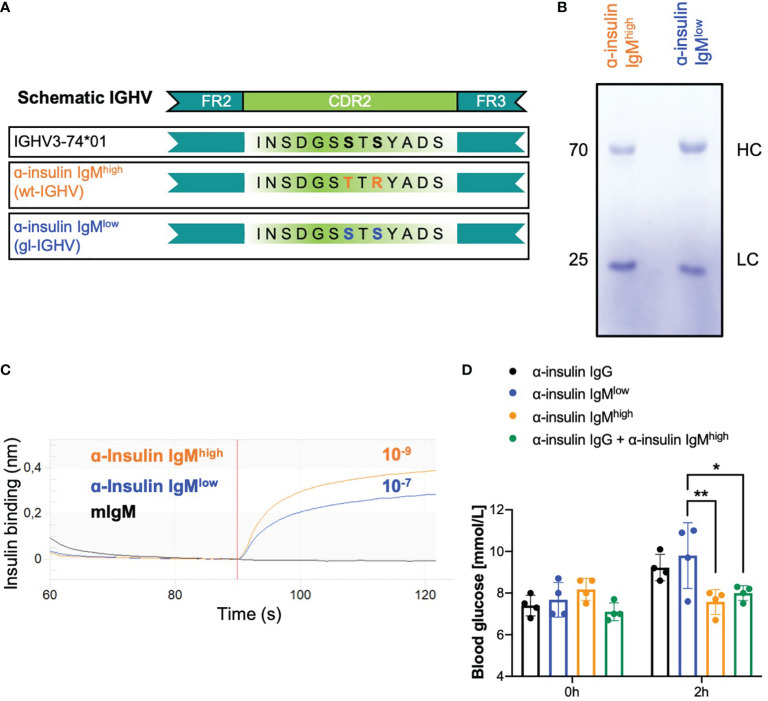
Recombinant low affinity anti-insulin IgM neutralizes insulin *in vivo.* **(A)** Schematic representation of the recombinant in-house purified anti-insulin IGHV highlighting the two mutations in the CDR2 which were reverted to the germline version of the IGHV3-74*01 allele. Orange: α-insulin IgM^high^ (WT-IGHV); blue: α-insulin IgM^low^ (gl-IGHV). **(B)** Coomassie-blue stained SDS-PAGE showing purified α-insulin IgM^high^ and α-insulin IgM^low^ under reducing conditions (with β-mercaptoethanol). The image is representative of three independent experiments. **(C)** Insulin-binding affinity of α-insulin IgM^high^ and α-insulin IgM^low^ measured by bio-layer interferometry. K_D_ (dissociation constant) was calculated by the software. The experiment shown is representative of 3 independent experiments. **(D)** Blood glucose concentrations of WT mice intravenously (i.v.) injected with 100 µg α-insulin IgG (black bar, n = 4), α-insulin IgM^low^ (blue bar, n = 4), α-insulin IgM^high^ (orange bar, n = 4) or α-insulin IgG + α-insulin IgM^high^ (n = 4, green bar) measured at indicated time points. Mean ± SD, statistical significance was calculated using two-way ANOVA with Sidak’s multiple comparisons test. *p < 0,05; **p < 0,01.

To confirm our hypothesis that IgM affinity and specificity determines the outcome of the interaction with the recognized cognate antigen, we used recombinant anti-insulin antibodies as model. In our previous study, we cloned a monoclonal anti-insulin antibody and studied its effect on insulin function when expressed as IgG or IgM isotype (18). This monoclonal antibody has high affinity (K_D_ 10^-9^) to its target autoantigen and neutralized insulin when expressed as IgG, while expression as IgM protected insulin from IgG-mediated neutralization *in vivo* (18). Since we proposed that affinity to the target and mono-specificity are the main requirements for determining the effector function of autoreactive antibodies, we expect that reversion of the variable region of the anti-insulin IgM into its respective germline (gl) version would result in reduced affinity to its target. To this end, we reverted the heavy chain (HC) and the light chain (LC) sequences to germline and tested combinations of the reverted HC/LC for their insulin binding affinity. While most combinations lost insulin binding, the recombinant insulin-specific antibody (anti-insulin IgM^low^) consisting of the original LC and the germline-reverted HC version of the anti-insulin antibody showed reduced affinity to insulin as compared with the original antibody (**Figure 1A**). In fact, the K_D_ of the germline-reverted anti-insulin IgM^low^ was in the range of 10^-7^ (**Figure 1C**) and thus, considerably lower than the affinity of the original anti-insulin IgM^high^. In addition, decreased insulin binding was observed for anti-insulin IgM^low^ by ELISA (**Supplementary Figure 1**). In order to test whether the two antibodies, namely anti-insulin IgM^low^ and its high affinity counterpart IgM^high^, had different effects on glucose metabolism, we injected identical molar amounts of anti-insulin IgM^high^ and anti-insulin IgM^low^ into WT mice. Within two hours after injections, higher blood glucose levels (hyperglycemia) were observed in mice that received anti-insulin IgM^low^, while anti-insulin IgM^high^ did not alter blood glucose and was able to protect insulin from IgG-dependent degradation (**Figure 1D**).

Interestingly, the reverted version of the anti-insulin IgM differs in only two point mutations in the complementarity-determining region 2 (CDR2) that seem to be responsible for the affinity maturation (**Figure 1A**). Importantly, the quality of the *in vitro* produced antibodies was assessed and revealed no structural difference between the purified IgM^high^ and the IgM^low^ antibodies (**Figure 1B**).

These data suggest that a high affinity autoantibody with a protective role can be turned into an autoantibody with a destructive role by reverting the immunoglobulin heavy chain variable region (IGHV) into its germline version (low affinity). This confirms our hypothesis of the regulatory role of IgM antibodies and suggests that mutations acquired during the affinity maturation process can turn destructive IgM antibodies into protective ones.

### High affinity RF enhances the effect of autoreactive IgG

**Figure 2:**
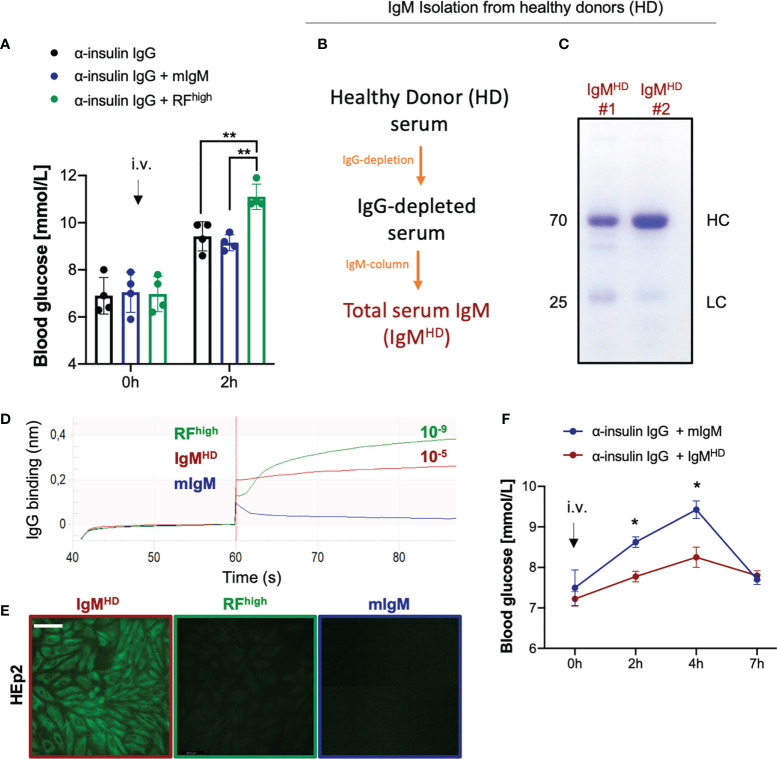
High affinity RF enhances the effect of autoreactive IgG. **(A)** Blood glucose concentrations of WT mice intravenously injected with 100 µg α-insulin IgG alone (black bar, n = 4) or in combination with 20 µg RF concentrate from rheumatoid arthritis patients (RF^high,^green bar, n = 4) or monoclonal IgM control (mIgM, blue bar, n = 4) measured at indicated time points. Mean ± SD, statistical significance was calculated using two-way ANOVA with Tukey’s multiple comparisons test. **p < 0,01. **(B)** Scheme depicting the procedure for isolation of total IgM from healthy donors (HD) sera. **(C)** Coomassie blue-stained SDS-PAGE showing total IgM isolation from n=2 healthy donors (IgM^HD^) under reducing conditions (with β-mercaptoethanol). The image is representative of three independent experiments. **(D)** IgG-binding affinity of IgM isolated from healthy donors (IgM^HD^, dark red line), RF^high^ (green line) and monoclonal IgM control (mIgM, blue line) measured by bio-layer interferometry. K_D_ (dissociation constant) was calculated by the software. The experiment shown is representative of 3 independent experiments. **(E)** HEp-2 slides showing anti-nuclear structure-reactive IgM (ANA) for total IgM isolation (IgM^HD,^dark red square), RF^high^ (green square) and monoclonal IgM control (mIgM, blue square). Scale bar 65 µm. Green fluorescence indicates IgM binding to HEp-2 cells. Images are representative of three independent experiments. **(F)** Blood glucose concentrations of WT mice intravenously (i.v.) injected with 100 µg α-insulin IgG combination with 20 µg total IgM purified from healthy donors (dark red line, n = 4) or with monoclonal IgM control (blue line, n = 4) measured at indicated time points. Mean ± SD, statistical significance was calculated using two-way ANOVA with Sidak’s multiple comparisons test. *p < 0,01.

Our previous data showed that anti-insulin IgG and anti-insulin IgM of different affinities are present in healthy individuals and contribute to the regulation of insulin function (18). In particular, injection of anti-insulin IgG isolated from intravenous immunoglobulin (IVIg) preparations in WT mice resulted in a significant increase in blood glucose (**Supplementary Figure 2A**) while the addition of high affinity anti-insulin IgM protected insulin from IgG-mediated degradation (18). To provide further evidence for the hypothesis that high affinity IgM protects its target antigen, we tested whether the protecting effect of high affinity IgM observed with the insulin-specific antibody also applies to other autoantibody:autoantigen combinations. To this end, we tested whether commercially available RF preparations isolated from RA patients act as protective IgM. As RF autoantibodies from RA patients typically bind with high affinity the Fc portion of IgG (14, 19, 24, 37), we refer to this RF as RF^high^. According to our proposed model (16–18), a high affinity PR-IgM autoantibody protects its target *in vivo* and therefore, we expected that RF^high^ protects and hence intensifies the effect of pathogenic IgG. To test this hypothesis, we co-injected anti-insulin IgG (from IVIg) together with RF^high^ or with a non-specific monoclonal control IgM (mIgM) into WT mice (**Figure 2A**). As already described (18), we observed increased blood glucose levels in animals that received the insulin-specific IgG which was comparable with the increase shown by mice that received the anti-insulin IgG along with mIgM. While the control mIgM showed no effect on the IgG-mediated change in blood glucose, animals co-injected with insulin-specific IgG and RF^high^ showed significantly higher blood glucose levels, suggesting that the RF^high^ binds to IgG and enhances its effect *in vivo.* To further confirm our hypothesis, we investigated whether RF having lower affinity for its target shows the opposite effect, and thus decreases the effect of anti-insulin IgG. Earlier studies have shown that anti-IgG IgM antibodies are not specific to RA patients as they can be detected in healthy individuals (19, 28, 29, 40). However, in contrast to RFs found in sera and synovia of RA patients exhibiting extensive somatic mutations and high affinity for their targets, RFs described in healthy donors are characterized by a limited number of mutations in their variable genes and low affinity to IgG (12, 19). We confirmed the presence of anti-IgG IgM in healthy donors and showed that they possess reduced affinity as compared with RF from RA patients (**Supplementary Figures 2B, C**). Since the amount of low affinity RF isolated from HD is not sufficient for injection experiments, we isolated total serum IgM from healthy donors (IgM^HD^) (**Figures 2B, C**) and confirmed by ELISA the presence of anti-IgG IgM antibodies (**Supplementary Figure 2D**). Using bio-layer interferometry (BLI), we assessed the IgG binding affinity of the eluted IgM^HD^ to be in the range of 10^-5^, thus lower than the affinity of RF^high^ (K_D_ 10^-9^) (**Figure 2D**). Furthermore, while RF^high^ displayed no reactivity in HEp2 slides, IgM^HD^ exhibited clear nuclear staining confirming available data showing that total IgM^HD^ from healthy donors is polyreactive (**Figure 2E**). To test whether the low affinity IgM^HD^ has a destructive effect *in vivo*, we injected WT mice with equal amounts of insulin-specific IgG in combination with identical amounts of control monoclonal IgM or IgM^HD^ containing low affinity RF. In contrast to the data obtained with RF^high^, animals injected with IgM^HD^ in combination with anti-insulin IgG exhibited normal glucose levels over the same range of time, indicative of the destructive effect of the low affinity RFs present in the IgM^HD^ elution (**Figure 2F**).

Altogether these data indicate that the regulatory roles of IgM possessing different affinities to its cognate antigen are not restricted to anti-insulin IgM antibodies and that low-affinity anti-IgG antibodies act as RF^low^ in healthy individuals thereby regulating IgG function.

### Recombinant low-affinity RF is polyreactive and binds DNA

**Figure 3:**
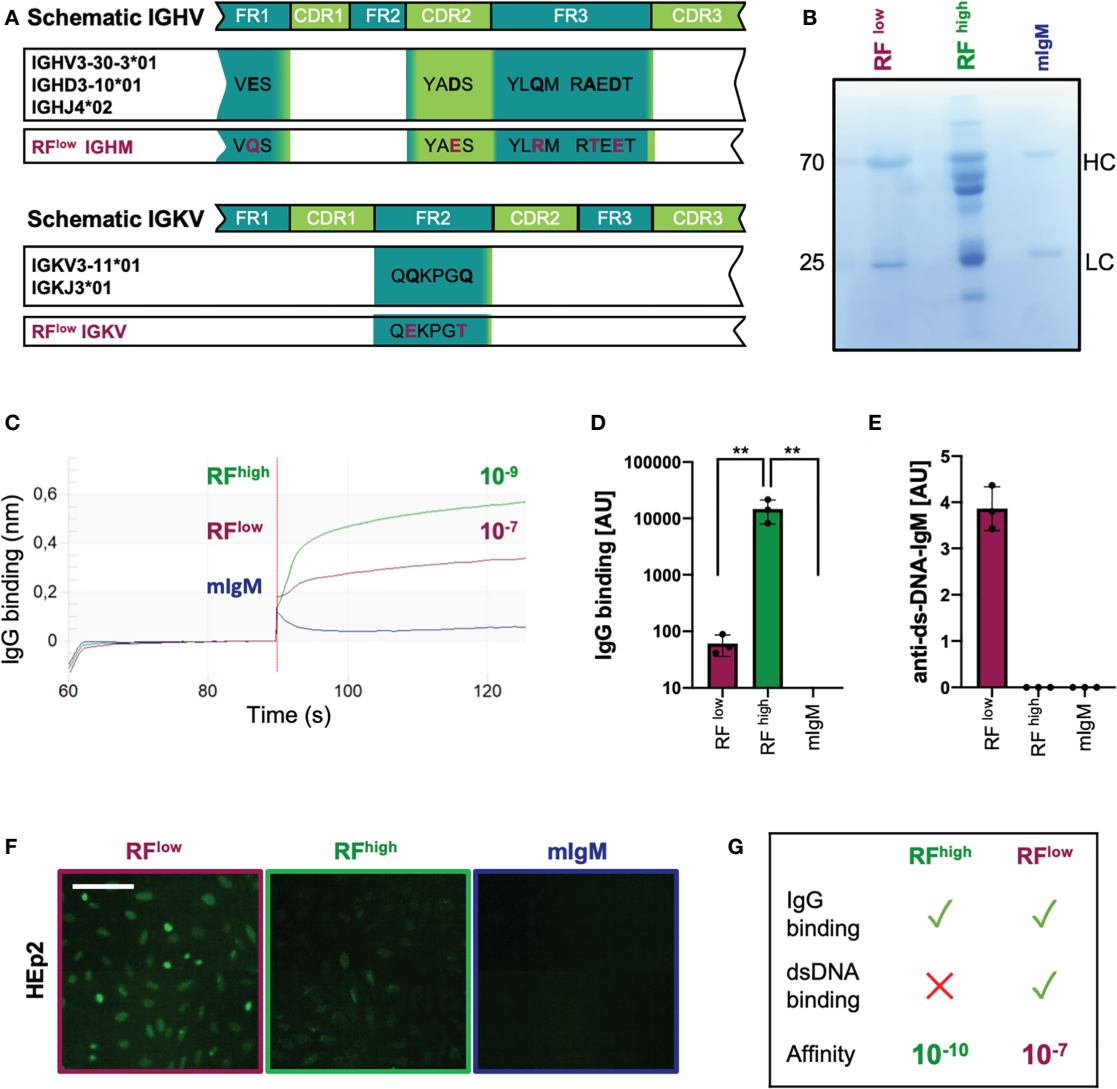
Recombinant low-affinity RF is polyreactive and binds DNA. **(A)** Schematic representation of the immunoglobulin heavy and light variable genes (IGHV and IGLV, respectively) of the recombinant purified low-affinity RF as compared to the closest germline respective alleles. Mutations are highlighted in purple. IGHM, immunoglobulin heavy constant mu; IGVK, immunoglobulin variable kappa. **(B)** Coomassie blue-stained SDS-PAGE showing recombinant monoclonal (in-house purified) low affinity RF (RF^low^), commercial RF from Rheumatoid Arthritis patients (RF^high^) and monoclonal control IgM (mIgM) under reducing conditions (with β-mercaptoethanol). The image is representative of three independent experiments. **(C)** IgG-binding affinity of RF^low^ (purple line), RF^high^ (green line) and monoclonal IgM (blue line) measured by bio-layer interferometry. K_D_ (dissociation constant) was calculated by the software. The experiment shown is representative of 3 independent experiments. **(D)** Anti-IgG IgM concentrations detected in purified RF^low^ (purple bar, n = 3), RF^high^ (green bar, n = 3) and mIgM control (blue bar, n=3) measured by ELISA (coating: human IgG). Mean ± SD, statistical significance was calculated using ordinary one-way ANOVA with Tukey’s multiple comparisons test. **p < 0,01. **(E)** Anti-dsDNA-IgM concentrations of RF^low^ (purple bar, n = 3), RF^high^ (green bar, n = 3) and IgM control (blue bar, n = 3) measured by ELISA (coating: calf-thymus dsDNA). Mean ± SD. Results are representative of three independent measurements. **(F)** HEp-2 slides showing anti-nuclear structure-reactive IgM (ANA). Purple square: RF^low^; green: RF^high^; blue: mIgM control, respectively. Scale bar 65 µm. Green fluorescence indicates IgM binding to HEp-2 cells. Images are representative of three independent experiments. **(G)** Schematic summary of the characteristics of RF^high^ and RF^low^.

In order to confirm our findings regarding the role of low affinity RF in the interaction with the target antigen, we reviewed available reports describing the extent of somatic mutations of RFs in RA patients (37, 41). Albeit the majority of RFs isolated from the synovia of RA patients are highly affine for the Fc portion of IgG and not reactive to other tested antigens, we identified one RF isolated from a RA patient that seemed to be polyreactive and bound to other antigens such as tetanus toxoid, DNA and bovine serum albumin (BSA) (37). Interestingly, in-depth analysis of the IGHV and IGLV sequences of the selected RF revealed a high degree of homology to the germline gene counterparts. In fact, the selected antibody variable heavy chain shared 96,9% identical residues with the IGHV3-30-3*01 (allele 1) and the light chain had 99,3% identity with the IGKV3-11*01 (**Figure 3A**). Due to the high degree of identity to germline genes and to the previously published data showing the polyreactivity of this RF, we expected this antibody to be low affinity RF (RF^low^). Therefore, we cloned and expressed RF^low^ as recombinant IgM (**Figure 3B**). Bio-layer interferometry assay revealed that the IgG-binding affinity of RF^low^ was in the range of 10^-7^, while the K_D_ of RF^high^ was 10^-9^ (**Figure 3C**).

The ability of RF^low^ to bind IgG was also tested by ELISA revealing that the recombinant RF^low^ binds IgG although to a lesser extent than RF^high^ which is most likely a result of the reduced IgG affinity of RF^low^ (**Figure 3D**). Additionally, we confirmed the previously published data showing that, in contrast to RF^high^, recombinant RF^low^ binds double-stranded DNA (**Figure 3E**) and is reactive in HEp2 slides (**Figure 3F**).

These data confirm available data suggesting that, in contrast to typical high affinity RFs from RA patients, low affinity RFs are multi-specific/poly-reactive as they bind DNA in addition to IgG (**Figure 3G**).

### RF^low^ controls IgG *in vivo* function by enhancing its degradation

**Figure 4:**
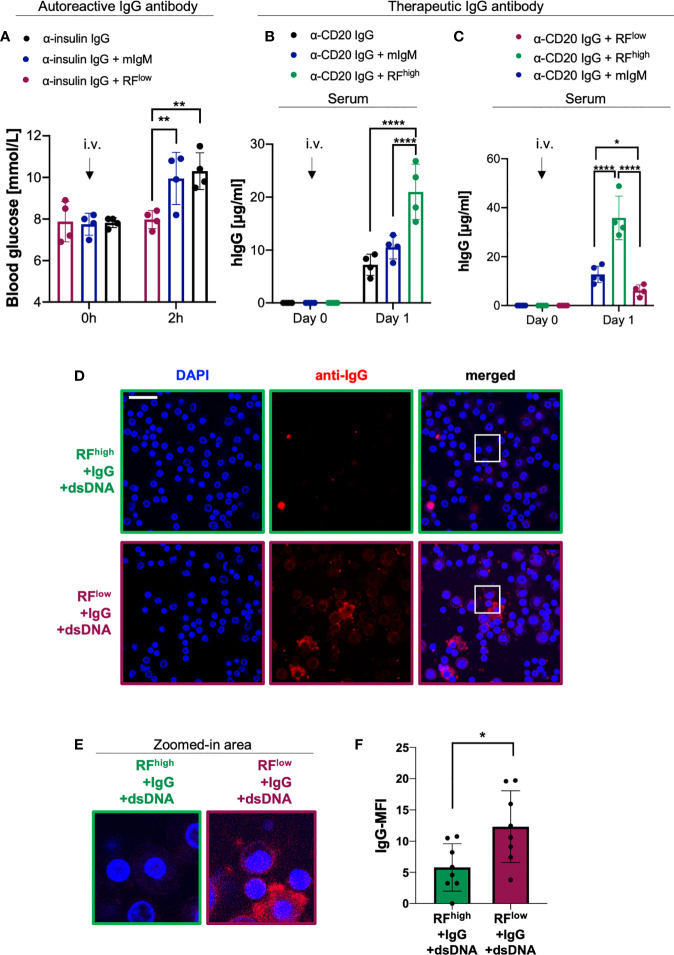
RF^low^ controls IgG *in vivo* function by enhancing its degradation. **(A)** Blood glucose concentrations of WT mice intravenously (i.v.) injected with 100 µg α-insulin IgG alone (black bar, n = 4) or in combination with 20 µg RF^low^ (purple bar, n = 4) or mIgM control (blue bar, n = 4) measured at indicated time points. Mean ± SD, statistical significance was calculated using two-way ANOVA with Tukey’s multiple comparisons test. **p < 0,01. **(B)** Serum human IgG concentrations of WT mice at day 0 and day 1 after a single i.v. injection of 20 µg of α-CD20 human IgG (rituximab) alone (black lined bar, n = 4) or in combination with RF^high^ (green lined bar, n=4) or mIgM control (blue lined bar, n = 4) as measured by ELISA. Mean ± SD, statistical significance was calculated using two-way ANOVA with Tukey’s multiple comparisons test. ****p<0,0001. **(C)** Serum human IgG concentrations of WT mice at day 0 and day 1 after a single i.v. injection of 20 µg α-CD20 human IgG (rituximab) in combination with RF^high^ (green line, n = 4), with RF^low^ (purple line, n = 4) or mIgM control (blue line, n = 5) as measured by ELISA. Mean ± SD, statistical significance was calculated using two-way ANOVA with Tukey’s multiple comparisons test. *p < 0,05; ****p < 0,0001. **(D)** Macrophage phagocytosis assays depicting the levels of macrophage immune complexes uptake. Macrophages were probed with complexes composed of RF^high^+IgG+dsDNA (top, green squared) or with complexes consisting of RF^low^+IgG+dsDNA (bottom, purple squared). Scale bar 29 µm. Images are representative of three independent experiments. **(E)** 4X Zoom-in of the white-squared areas in **Figure 4D** as indicated above each image. **(F)** Quantification of mean fluorescent intensity (MFI) of IgG phagocytosed by macrophages detected using anti-IgG antibody: RF^high^+IgG+dsDNA (green bar), RF^low^+IgG+dsDNA (purple bar). Data were normalized to IgG-MFI of macrophages probed with immune complexes composed of IgG and dsDNA (shown in **Supplementary Figure 4**). Dots represent single cells (n = 8). Mean ± SD, statistical significance was calculated using unpaired t test with Welch’s correction. *p < 0,05.

Using the above described monoclonal RF^low^, we tested whether a low affinity RF would neutralize its target *in vivo.* To this end, we injected WT mice with anti-insulin IgG together with equal molar amounts of RF^low^ or with a non-specific monoclonal IgM as control (mIgM). As expected, mice injected with anti-insulin IgG only or with anti-insulin IgG together with control mIgM showed comparable increase in blood glucose levels. In contrast, mice which received RF^low^ along with the anti-insulin IgG showed constant blood glucose level suggesting that RF^low^ controls the function of autoreactive IgG (**Figure 4A**).

Next, we investigated whether the protective or destructive effect of RF could be observed with other IgGs such as therapeutic antibodies. To this end, we used rituximab as a well-known therapeutic IgG antibody targeting CD20. This monoclonal anti-CD20 antibody, which consists of human constant regions and murine variable domains (42, 43), is approved for treatment of B cell malignancies as well as autoimmune diseases such as RA and ANCA-associated vasculitis (14, 27, 44, 45). We intravenously injected into WT mice equal molar amounts of anti-CD20 IgG either alone or combined with RF^high^ or with mIgM and we monitored human IgG (hIgG) concentrations over time. Our data showed that mice injected with rituximab together with RF^high^ exhibited significantly higher levels of hIgG as compared to mice that received anti-CD20 IgG alone or in combination with mIgM (**Figure 4B**). Importantly, barely detectable traces of IgG in the commercial RF^high^ preparation are unlikely to affect the experimental set-up as shown by the significantly lower IgG levels detected when RF^high^ was injected alone (**Supplementary Figure 3A**). Interestingly, the presence of RF^high^ stabilized hIgG in sera up to 7 days post injection, while the addition of mIgM to anti-CD20 IgG did not show any significant change in hIgG concentration as compared to the hIgG levels of mice that received anti-CD20 IgG only (**Supplementary Figure 3B**). Together with the data above, these results led us to the hypothesis that if the higher hIgG titer was due to the presence of RF^high^, the co-injection of RF^low^ with anti-CD20 IgG should show opposite effect, i.e. reduction of hIgG concentrations over time. Therefore, we injected anti-CD20 IgG combined with equal molar amounts of the recombinant RF^low^ or of the control monoclonal mIgM. We observed a significant difference in the hIgG level between the two groups already one day after injection. In fact, the animals injected with RF^low^ along with anti-CD20 IgG showed significantly lower concentrations of hIgG in respect to the mice which received RF^high^ (**Figure 4C**). Our previous work (18) suggested that the ability of RF^low^ to simultaneously bind its target and dsDNA could facilitate the formation of large immune complexes (ICs) and, consequently, uptake by macrophages, which are known to phagocyte IgM ICs (46–49). To confirm this hypothesis, we incubated RF^high^ or RF^low^ with IgG and genomic dsDNA to generate corresponding ICs. Interestingly, ICs consisting of RF^low^ and dsDNA with IgG exhibited higher rates of uptake by macrophages as compared to ICs formed by RF^high^ and dsDNA with IgG (**Figures 4D-F** and **Supplementary Figure 4**). To further corroborate these data, we performed an analogous experiment by incubating insulin with anti-insulin IgM^high^ or anti-insulin IgM^low^ in the presence of genomic dsDNA allowing ICs formation. As expected, ICs formed by anti-insulin IgM^low^ showed increased phagocytic uptake as compared with ICs formed by anti-insulin IgM^high^ (**Supplementary Figures 5A, B**).

Our results show that a high affinity RF is capable of stabilizing IgG *in vivo* thereby extending IgG half-life, while a low affinity RF exhibit the opposite destructive effect on IgG *in vivo.* Importantly, this effect is due to the ability of polyreactive low affinity RF (RF^low^) to form immune complexes which are prone to be taken up by macrophages. Together, these data indicate that RFs have different impact on the half-life of IgG depending on their affinity to their target. Interestingly, this is not only valid for autoreactive antibodies but also for therapeutic antibodies.

### RF^low^ prevails over RF^high^

**Figure 5:**
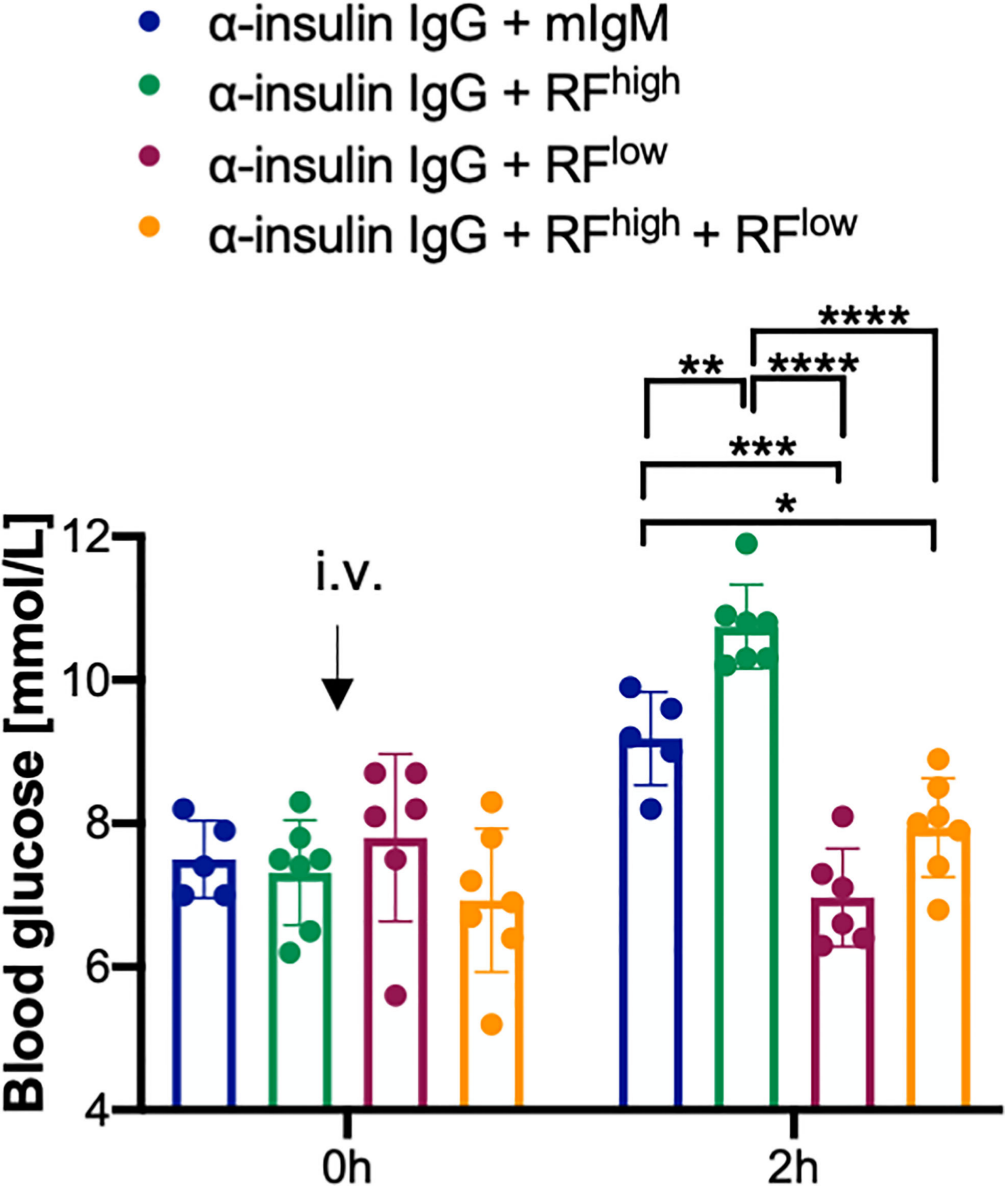
RF^low^ prevails over RF^high^. Blood glucose concentrations of WT mice intravenously (i.v.) injected with 100 µg α-insulin IgG in combination with 20 µg RF^high^ (green bar, n = 7), 20 µg RF^low^ (purple bar, n = 6), 20 µg RF^low +^ 20 µg RF^high^ (orange bar, n = 7) or mIgM control (blue bar, n = 5) measured at indicated time points. Mean ± SD, statistical significance was calculated using two-way ANOVA with Tukey’s multiple comparisons test. *p < 0,05; **p < 0,01; ***p < 0,001; ****p < 0,0001.

In order to better understand the dynamic of RFs interaction with IgG *in vivo*, we investigated the effects of the combined presence of low and high affinity RFs on IgG function. To this end, we injected into WT mice anti-insulin IgG with equal molar amounts of RF^high^ and RF^low^ and subsequently, we monitored blood glucose levels. As expected, the blood glucose levels of the mice injected with anti-insulin IgG combined with mIgM were increased within two hours after injection. Intriguingly, the blood glucose levels of the mice injected with the insulin-specific IgG and the combination of RF^high^ and RF^low^ (anti-insulin IgG + RF^high^ + RF^low^) did not differ from the levels of mice injected with anti-insulin IgG with RF^low^ only, suggesting that RF^high^ cannot exert its protective role in the presence of RF^low^. In fact, blood glucose concentrations in these mice, which received anti-insulin IgG + RF^high^ + RF^low^, were significantly lower than the concentrations in mice which received anti-insulin IgG with RF^high^ alone (**Figure 5**).

In summary, our data suggest that the presence of RF^low^ counteracts the stabilizing activity of RF^high^ resulting in target destruction which is comparable to the effect observed with RF^low^ only.

### Deregulated ratios of high affinity and low affinity RFs in autoimmune diseases

**Figure 6:**
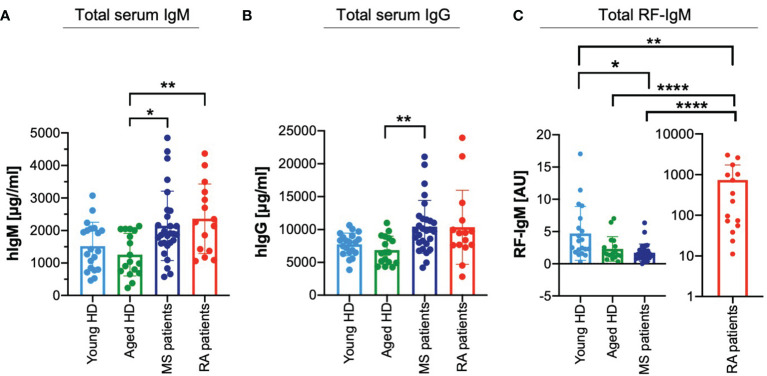
Deregulated ratios of high affinity and low affinity RFs in autoimmune diseases. **(A)** Total IgM amount detected in serum from young (light blue bar, n = 20) and aged (green bar, n = 17) healthy donors (HD), multiple sclerosis (MS) patients (blue bar, n = 28) and rheumatoid arthritis (RA) patients (red bar, n = 15) as measured by ELISA. Bars depict mean ± SD, individual values are represented by single dots. Statistical significance was calculated using Kruskal Wallis test. *p < 0,05; **p < 0,01. Mean values of IgM (μg/ml) as follows: young HD 1517.55; aged HD 1258.02; MS patients 2143.72; RA patients 2361.29. **(B)** Total IgG amount detected in serum from young (light blue bar, n = 20) and aged (green bar, n = 17) healthy donors (HD), multiple sclerosis (MS) patients (blue bar, n = 28) and rheumatoid arthritis (RA) patients (red bar, n = 15) as measured by ELISA. Bars depict mean ± SD, individual values are represented by single dots. Statistical significance was calculated using Kruskal Wallis test. ** p < 0,01. Mean values of IgG (μg/ml) as follows: young HD 7733.22; aged HD 6856.48; MS patients 10419.28; RA patients 10345.23. **(C)** Total RF-IgM amount detected in serum from young (light blue bar, n = 20) and aged (green bar, n = 17) healthy donors (HD), multiple sclerosis (MS) patients (blue bar, n = 28) and rheumatoid arthritis (RA) patients (red bar, n = 15) as measured by ELISA. (coating: human IgG). Bars depict mean ± SD, individual values are represented by single dots. Values from RA patients plotted separately for simplified visualization. Statistical significance was calculated using Kruskal Wallis test. *p < 0,05; **p < 0,01; ****p < 0,0001. Mean values of RF-IgM (AU) as follows: young HD 4.71; aged HD 2.31; MS patients 1.72; RA patients 737.58.

The above results suggesting that the effects observed in the presence of a low affinity RF prevails over the effects of a high affinity RF lead us to the hypothesis that a failure in maintaining the balance between the two classes of RFs might contribute to the development of autoimmune diseases. To gain a deeper understanding, we collected sera from young and aged healthy donors and from patients suffering from two well-known autoimmune diseases, namely RA and multiple sclerosis (MS). We characterized these samples for total serum levels of IgM and IgG. Interestingly, total serum IgM levels of MS and RA patients seem to be increased as compared with healthy individuals (**Figure 6A**). Furthermore, while total serum IgG concentrations of young and aged healthy individuals were in a similar range, the IgG levels of MS patients were significantly increased as compared to aged healthy individuals and a similar, although not significant, tendency was shown by total IgG levels of RA patients (**Figure 6B**).

Next, we assessed whether the higher circulating levels of IgG in MS correlate with altered amounts of circulating RF-IgM. Interestingly, MS patients showed significantly lower amounts of RF-IgM than the young and aged healthy individuals (**Figure 6C**). These data suggest that low affinity RF is reduced in MS patients as compared with healthy individuals and, therefore, it is conceivable that the regulation of IgG homeostasis including autoreactive antibodies is altered.

Altogether, these findings suggest that an increase in IgG-protective RF^high^ in RA patients or a decrease in IgG-destructive RF^low^ in MS patients might be important pathogenic mechanisms associated with the development of autoimmune diseases.

## Discussion

Although being one of the best characterized autoantibodies, the role of RF-IgM in RA disease pathogenesis remains elusive. Several studies pointed out that these antibodies are produced in response to increasing level of autoreactive IgG (e.g. ACPA autoantibodies) and that the formation of immune complexes further enhances the inflammatory process in the synovia (14, 20, 24, 27).

Using a pathogenic autoreactive IgG as well as a therapeutic IgG, we propose in this study a novel role for anti-IgG IgMs and suggest that they act as regulators of IgG turnover. Compliant to our previous results describing the existence of a protective regulatory IgM (PR-IgM) (17, 18) as important control of physiological homeostasis, this study shows that high affinity RFs stabilize IgG and intensify its effect *in vivo*. On the other hand, we describe for the first time that low affinity RFs display opposite neutralizing effects on IgG, thereby leading to faster degradation and diminishing of IgG *in vivo*. These effector functions are merely dependent on the affinity of RF-IgM for IgG and independent of the pathogenic or beneficial nature of the target IgG. In fact, we show that high affinity RF stabilizes the autoreactive insulin-specific IgG, which was earlier described as regulator of insulin homeostasis (16–18), and induces higher blood glucose levels prolonging the hyperglycemic state. On the contrary, we demonstrate that low affinity RF neutralizes the autoreactive insulin-specific IgG thereby preventing the drastic increase in blood glucose. Similarly, RFs exert a stabilizing role when they are monospecific and possess high affinity for a therapeutic IgG, while they contribute to faster degradation if their affinity to IgG is low and if they are polyreactive. The fact that low affinity RFs are found in healthy individuals and regulate half-life of IgG suggests that IgG homeostasis is controlled by such RFs and that defects in generating RF^low^ might be an important trigger for the development of autoimmune diseases. In this scenario, we here show that polyreactive, low-affinity RF antibodies neutralize IgG antibodies by forming large ICs together with nucleic acids thereby facilitating IgG uptake by phagocytes. Importantly, low affinity RFs might act as general regulators of IgG by recognizing its constant region. Alternatively, low affinity RFs might act in a distinctive manner by regulating specific IgG idiotypes through the recognition of the individual variable region (50). In the context of IgG-associated autoimmune diseases, this suggests that a highly diverse antibody repertoire is important for the regulation of large spectrum of IgG antibodies targeting individual idiotypes.

This scenario is in sharp contrast to the current view proposing that autoantibodies develop in consequence to defects in central and peripheral tolerance mechanisms which in healthy conditions should prevent the development of autoreactive B cells (4–7, 9, 11, 12, 15, 51). As such, it is hard to explain the presence of autoantibodies such as RFs in normal population purely as a result of defective tolerance. In fact, we assume that B cells with autoreactive specificities might be positively selected during their development because they possess important regulatory role in homeostasis and immune reactions against different kind of antigens (35). In line with this view, the existence of autoantibodies in healthy population points towards the existence of physiological autoimmunity (36). Notably, the absence of secreted autoreactive natural IgM has been correlated with accelerated development of IgG autoantibodies in lupus-prone lymphoproliferative (lpr) mouse model, confirming the physiological relevance of natural IgM autoantibodies in ameliorating IgG-associated autoimmune pathology (52). Thus, autoimmunity is most likely central for physiological homeostasis and should not be confused with autoimmune diseases. As such, not all autoreactive B cells are pathogenic and additional elements including genetic and environmental factors, would be required for the development of autoimmune diseases (53, 54). Similarly, although RFs are mostly associated with RA, several studies have shown that they can also be found in sera of healthy individuals suggesting that their presence does not necessarily lead to disease pathogenesis (12, 19, 20, 28, 29).

Remarkably, RFs found in RA significantly differ from RFs found in healthy individuals as the latter are polyreactive and show little signs of affinity maturation (12, 19, 32, 33). Conversely, RFs expressed by RA patients are highly somatically mutated, monospecific and possess high affinity for IgG (12, 19, 20, 31, 37, 41). In line with this view, it is tempting to speculate that the borderline between physiologic and pathologic autoimmunity is strongly marked by the affinity to the autoantigen rather than solely by tolerance mechanisms. Based on our data with insulin, it is conceivable that high affinity and low affinity IgM antibodies with opposite effects on their cognate antigen can develop against practically every autoantigen. Defects in establishing these equilibria may most likely lead to the development of autoimmune responses.

In line with this scenario, it is feasible that the high titers of high affinity RFs in the synovial membrane of RA patients acquires pathogenic role because they perpetuate the inflammatory state by stabilizing pathogenic IgG such as ACPA. The exaggerated function of autoreactive IgG in the joints leads to the formation of immune complexes that may continuously trigger macrophages and complement activation *via* Fc receptors thereby prolonging inflammation at the synovial membrane. Importantly, the chronic inflammation associated with increased stability of pathogenic ACPA-IgG might also contribute to the extra-articular involvement in RA patients, including cardiovascular comorbidities such as impaired cardiac function, accelerated atherosclerosis and endothelial dysfunction (55, 56).

On the contrary, low affinity destructive RFs may control the general IgG homeostasis when they recognize the constant region of IgG, whereas they may selectively eliminate pathogenic IgGs when acting at the level of individual idiotypes. Characterizing individual RFs regulating pathogenic or therapeutic antibodies has a great potential for the treatment of IgG-mediated immune diseases and for improving the efficacy of therapeutic antibodies.

Interestingly, low affinity RFs resemble the so-called natural autoantibodies present in healthy population. These natural IgM autoantibodies are involved in the clearance of defective self-structures and have already been suggested to play a homeostatic role in the regulation of inflammation by ameliorating the phenotype induced by destructive autoimmunity (32, 33, 52, 57–59). Based on the limited mutation rate and the reduced affinity, we propose that natural autoantibodies are primary IgM antibodies that are secreted in the course of early B cell activation before affinity maturation. In fact, earlier studies have shown that despite a similar use of V light and V heavy genes, RFs in healthy individuals show significantly reduced mutation patterns in their CDRs as compared with RFs of RA patients (19, 60–62). In full agreement, several evidences pointed out that anti-IgG secreting B cells (RF-secreting B cells) are commonly found in humans and may play a role in antigen presentation and regulation of immune response (30, 63). Furthermore, it has been reported that RF-B cells are commonly generated in mice and humans during the course of secondary immune response to lipopolysaccharide antigens and immune complexes (64–66). Notably, sequencing analysis revealed that these RFs are encoded by a highly similar and limited range of germline genes, thus being of low affinity. These RFs may have beneficial physiological and immunoregulatory effects, contributing to clearance of the target antigen (66, 67). In light of this view, the low affinity RFs found in healthy population are most likely the result of regulated selection mechanisms that limit affinity maturation in healthy individuals thereby preventing low affinity RF autoantibodies from becoming pathogenic (19, 33).

An important conclusion of our study is that in the simultaneous presence of high and low affinity RFs, the effect of the destructive low affinity RF prevails over the protective high affinity RF. Extending this finding to a more general level, it is conceivable that the ratio between the two RF populations is relevant in the context of autoimmunity.

Here, we show that patients affected by autoimmune diseases have somewhat higher levels of total serum IgM and IgG antibodies as compared to healthy donors. However, the amount of RF-IgM detected in MS patients is significantly lower than the amount observed in healthy individuals. Thus, while RA patients are characterized by elevated amounts of high affinity protective RFs resulting in escalation of IgG function including autoreactive antibodies, MS patients most likely lack low affinity destructive RFs. The absence of low affinity RFs results in altered hemostasis of IgG antibodies leading to the accumulation and intensification of IgG function including autoreactive specificities.

Taken together, this study proposes RF-IgMs as regulators of IgG homeostasis and correlates the effects exerted by RF autoantibodies to their binding-affinity to the target IgG. This includes the existence of polyreactive neutralizing IgM as opposed to the PR-IgM (16–18). These findings indicate that one way to possibly reduce the levels of harmful IgG autoantibodies in circulation would be the use of low affinity RF or total IgM antibodies from healthy individuals as therapeutic antibodies. Interestingly, the generation of idiotype-specific anti-IgG IgM would allow the manipulation of individual IgGs in a specific manner without affecting the entire IgG repertoire.

Collectively, our findings pave the way to a better understanding of immune regulation and open up new opportunities for the development of novel therapies.

## Data availability statement

The original contributions presented in the study are included in the article/**Supplementary Material**. Further inquiries can be directed to the corresponding author.

## Ethics statement

The animal study was reviewed and approved by Regierungspräsidium Tübingen (license 1484), Baden-Württemberg, Germany. Biobanking of RA patients’ samples was approved by the ethical committee of the University of Freiburg (ethical vote No 507/16). All human blood samples were collected and processed according to ethics guidelines approved by Ulm University Ethics Committee (ethical vote 385/21). Written informed consent for participation was not required for this study in accordance with the national legislation and the institutional requirements.

## Author contributions

AN and TA planned, performed and analyzed experiments, interpreted data, and contributed to writing of the manuscript. OEA and MY performed experiments. SF, MS and REV provided RA and MS patient samples and contributed to writing the manuscript. HJ designed the experiments, wrote the manuscript, and supervised the study. All authors contributed to the article and approved the submitted version.

## Funding

This work was supported by the DFG through TRR130 (project 01 and 12), SFB1279 (project B03) and SFB 1506 (project B05).

## Acknowledgments

The authors are thankful to Gabriele Allies for excellent technical assistance. We thank Theresa Schleyer for expert assistance with biobanking. We thank Dr. Christian Bökel from the Core Facility “Confocal and Multiphoton Microscopy” (Faculty of Medicine-Ulm University) for expert technical assistance with microscopy.

## Conflict of interest

The authors declare that the research was conducted in the absence of any commercial or financial relationships that could be construed as a potential conflict of interest.

## Publisher’s note

All claims expressed in this article are solely those of the authors and do not necessarily represent those of their affiliated organizations, or those of the publisher, the editors and the reviewers. Any product that may be evaluated in this article, or claim that may be made by its manufacturer, is not guaranteed or endorsed by the publisher.
