# Erratum: Rheumatoid factor IgM autoantibodies control IgG homeostasis

**DOI:** 10.3389/fimmu.2022.1123117

**Published:** 2022-12-29

**Authors:** Frontiers Production Office

**Affiliations:** Frontiers Media SA, Lausanne, Switzerland

**Keywords:** autoantibodies, rheumatoid factor, affinity, homeostasis, autoimmunity

Due to a production error, an author’s name was incorrectly spelled as “Omal El Ayoubi “. The correct spelling is “Omar El Ayoubi”. The publisher apologizes for this mistake.

The original version of this article has been updated.

