# Supplementary material for: Rheumatoid factor IgM autoantibodies control IgG homeostasis
Source: Front Immunol. 2022 Oct 21;13:1016263. doi: 10.3389/fimmu.2022.1016263 (PMC9634112; doi:10.3389/fimmu.2022.1016263)
Supplement: Supplementary file 1 [file Presentation_1.pdf]

Supplementary Figure 1

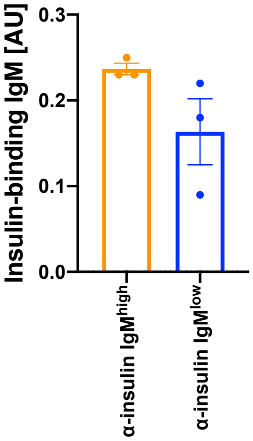

Supplementary Figure 1
Anti-insulin-IgM concentrations detected in recombinant in-house purified α-insulin IgM^{high} (WT-IGHV, orange bar, n=3) and α-insulin IgM^{low} (gl-IGHV, blue bar, n=3) as measured by ELISA (coating: human insulin). Mean ± SD depicted. Data are representative of three independent measurements**.**

Supplementary Figure 2

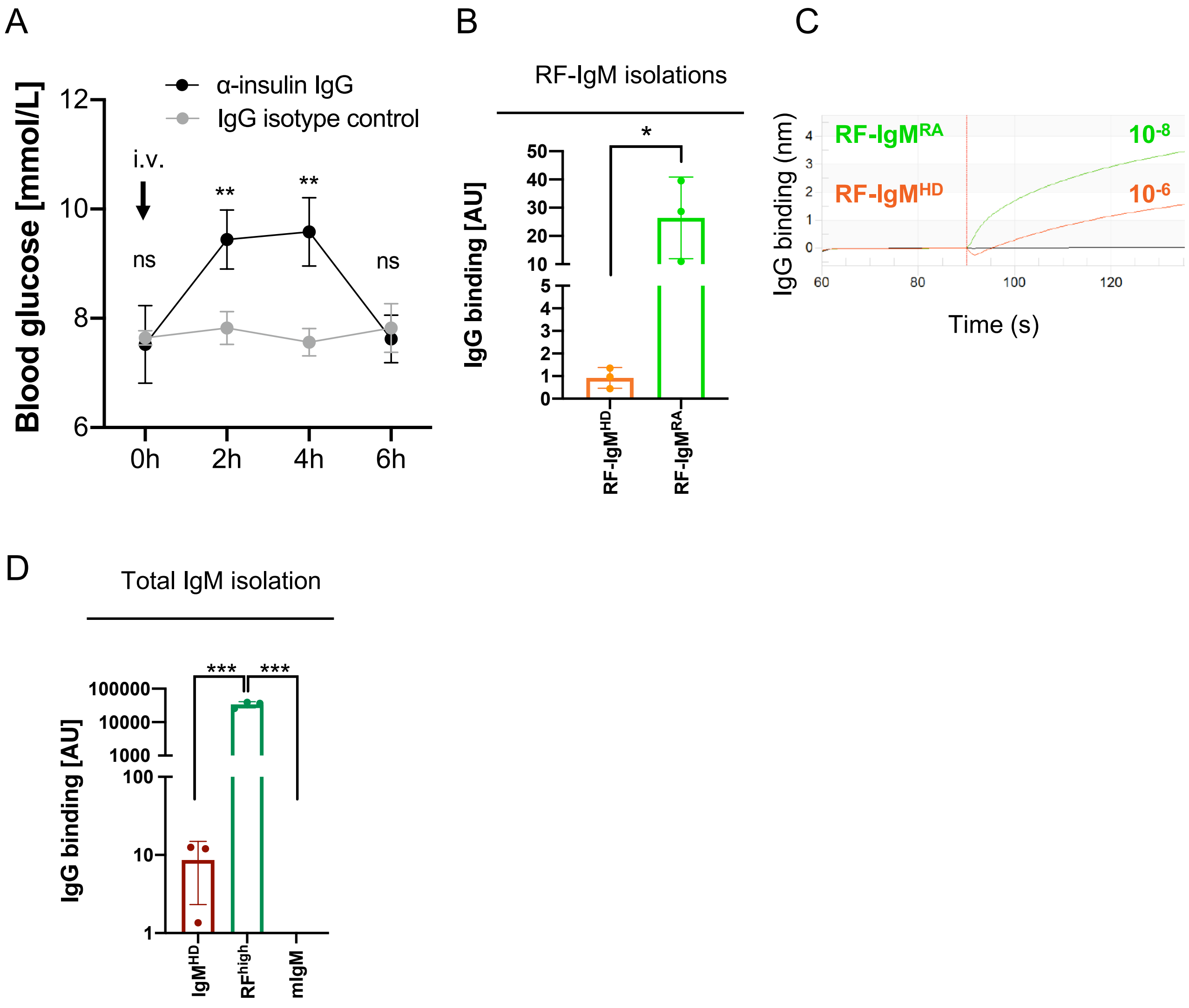

Supplementary Figure 2

A Kinetic plot showing mean ± SD of blood glucose levels after injection of 100 µg α-insulin IgG (black line, n=5) or IgG isotype control (grey line, n=5). Statistical significance was calculated using two-way ANOVA with Sidak's multiple comparisons test. ** p<0,01

B IgG-binding IgM concentrations in RF-IgM elution from healthy donors (RF-IgM^{HD,} orange bar, n=3) and from RA patients (RF-IgM^{RA}, green bar, n=3) as measured by ELISA (coating: human IgG). Mean ± SD, statistical significance was calculated using unpaired t test. * p<0,05.

C IgG-binding affinity of RF-IgM isolated from healthy donors (RF-IgM^{HD}, orange line) and from RA patients (RF-IgM^{RA}, green line) measured by bio-layer interferometry. K_D (dissociation constant) was calculated by the software. The experiment shown is representative of 3 independent experiments.

D IgG-binding IgM concentrations in total IgM isolated from healthy donors (dark red bar, n=3) compared with IgG-binding IgM amount detected in RF^{high} (green bar, n=3) and in monoclonal IgM (n=3) as measured by ELISA (coating: human IgG). Mean ± SD, statistical significance was calculated using ordinary one-way ANOVA with Tukey's multiple comparisons test. *** p<0,001

Supplementary Figure 3

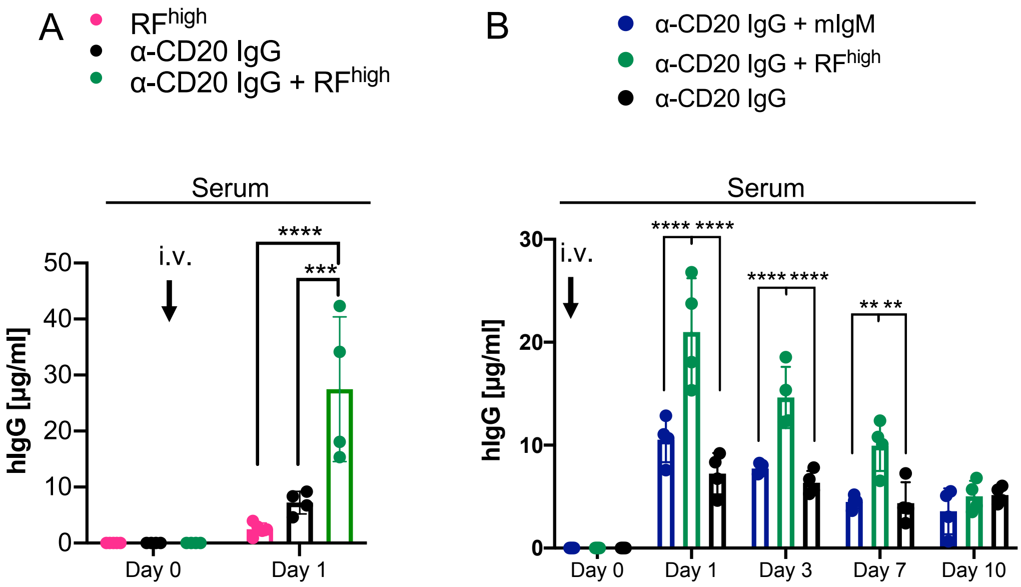

Supplementary Figure 3

A Serum human IgG concentrations of WT mice after a single i.v injection of 20 µg RF^{high} alone (pink bar, n=5), 20 µg α-CD20 IgG alone (black bar, n=4) or 20 µg α-CD20 IgG+ RF^{high} (green bar, n=4). Mean ± SD, statistical significance was calculated using two-way ANOVA with Sidak's multiple comparisons test. *** p<0,001, **** p<0,0001

B Serum human IgG concentrations of WT mice after a single i.v injection (day 0) of 20 µg α-CD20 IgG (rituximab) alone (black bar, n=4) or in combination with 20 µg RF^{high} (green bar, n=4) or 20 µg mIgM ctrl (blue bar, n=4) at indicated time points. Mean ± SD, statistical significance was calculated using two-way ANOVA with Tukey's multiple comparisons test ** p<0,01; **** p< 0,0001.

Supplementary Figure 4

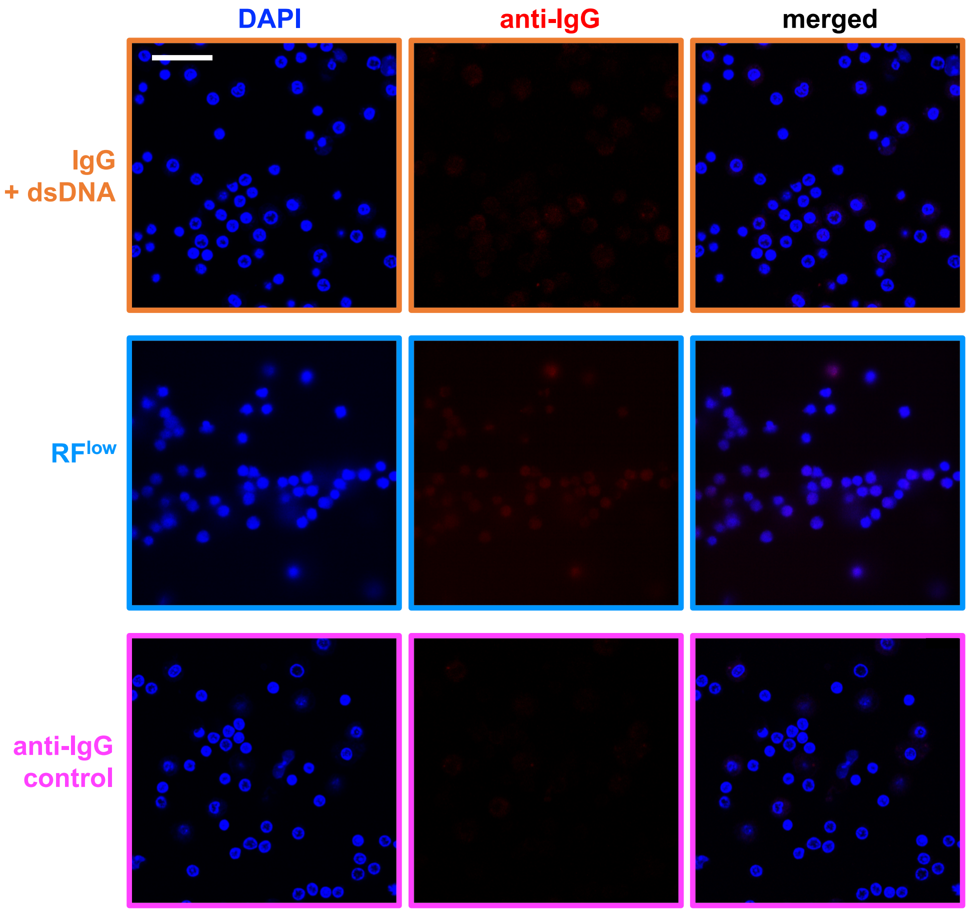

Supplementary Figure 4

Macrophage phagocytosis assays depicting the levels of macrophage immune complexes uptake. As control, macrophages were probed with complexes composed of IgG+dsDNA (top panel, orange squared). Alternatively, cells incubated with RF^{low} only were used as a control to rule out that the polyreactivity of RF^{low} enables its binding to the cells as well as to the anti-IgG detection antibody (middle panel, light blue squared). In addition, macrophages were left untreated and anti-IgG detection antibody was used alone to exclude its cross reactivity with the cells (bottom panel, pink squared). Scale bar 29 µm. Images are representative of three independent experiments.

Supplementary Figure 5

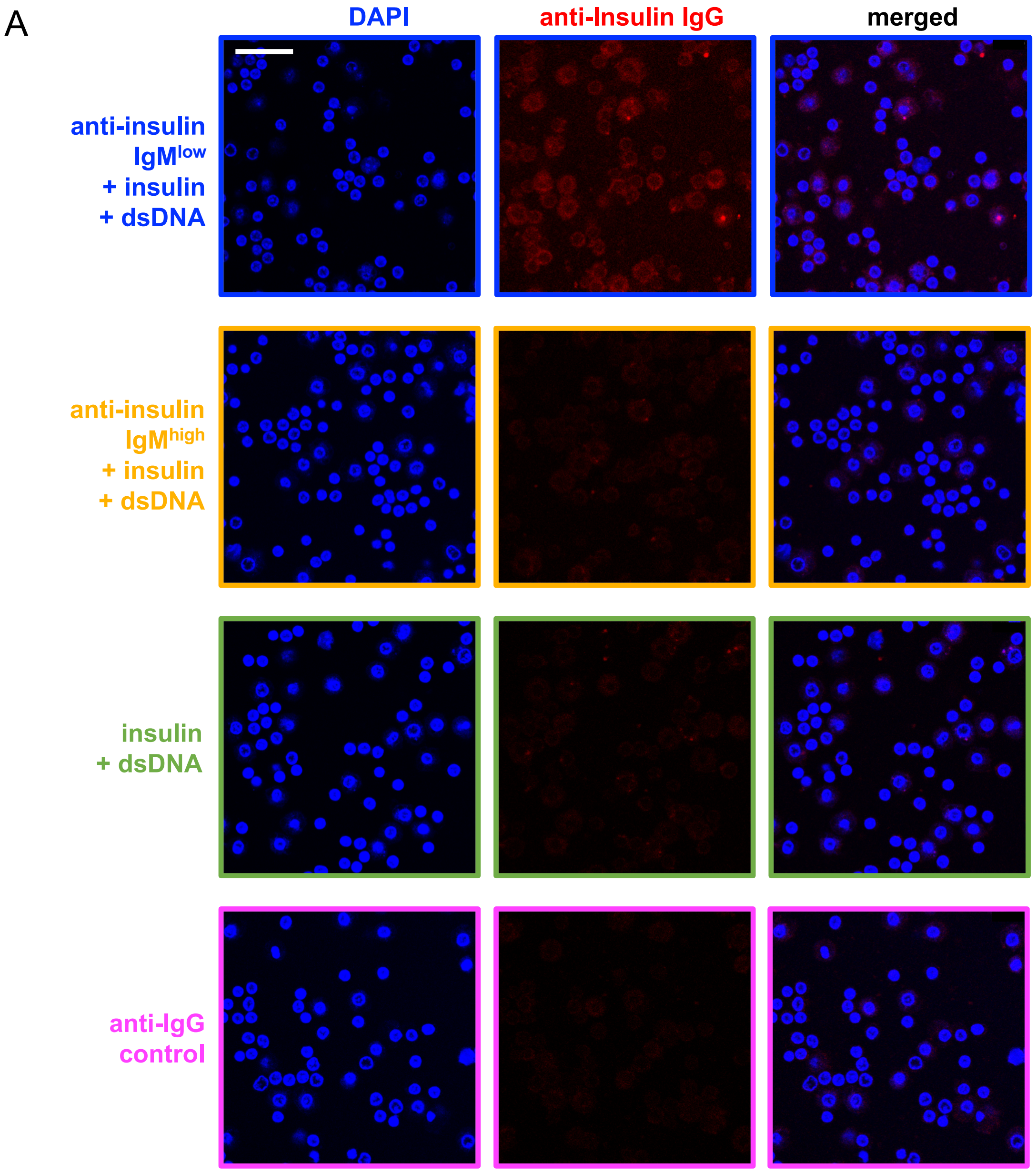

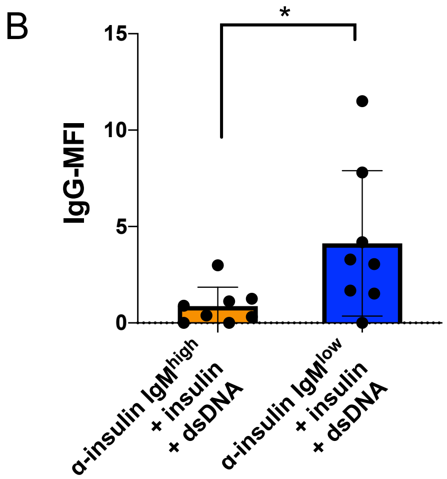

Supplementary Figure 5

A Macrophage phagocytosis assays depicting the levels of macrophage immune complexes uptake. Macrophages were probed with complexes composed of ɑ-insulin IgM^{low}+insulin+dsDNA (blue squared), ɑ-insulin IgM^{high}+insulin+dsDNA (orange squared), insulin+dsDNA (green squared) or ɑ-insulin IgG control (pink squared). Scale bar 29 µm. Images are representative of three independent experiments.

E Quantification of mean fluorescent intensity (MFI) of insulin phagocytosed by macrophages detected via ɑ-insulin IgG: ɑ-insulin IgM^{high}+insulin+dsDNA (orange bar), ɑ-insulin IgM^{low}+insulin+dsDNA (blue bar). Data were normalized to IgG-MFI of macrophages probed with immune complexes composed of insulin and dsDNA. Dots represent single cells (n=8). Mean ± SD, statistical significance was calculated using unpaired t test with Welch's correction. *p<0,05
